# PB.39: Minimising the impact of breast screening extension: a 1-year experience of a South West breast screening unit

**DOI:** 10.1186/bcr3539

**Published:** 2013-11-08

**Authors:** K Giles, R Currie

**Affiliations:** 1 Royal Devon and Exeter Hospital, Exeter, UK; 2 Exeter and North Devon Breast Screening Unit, Exeter, UK

## Introduction

In 2007, the Cancer Reform Strategy announced an extension to the NHS Breast Screening Programme (NHSBSP) from women aged 50 to 70 to those 47 to 73 years. Pilot studies involved a 50% randomised cohort at either the upper or lower end of the extension. We detail how our BSU has instigated the complete lower age extension largely within existing capacity.

## Methods

Data collection from NBSS. Working practices identified from local policy.

## Results

Screening numbers increased from 22,022 to 26,272 (a 19% increase), generating an extra 355.2 screening hours. This constituted a 76% invite uptake in women aged 47 to 49. Screening appointments were reduced from 6 to 5 minutes. Mammographers working patterns - but not overall hours - were changed. An extra workstation was purchased for additional film reading by existing personnel. All assessment clinics became fully booked sessions (recall rate 9.4%). Biopsies were largely undertaken in existing capacity but provision was increased when needed by altering staff working patterns. Women requiring MDT discussion led to a marginal increase in MDT length. All biopsy results were delivered by breast care nurses - the extra work load required an additional session (4 hours) to fulfil the demand. All screening targets were met.

## Conclusion

Full lower end age extension has undoubtedly increased our screening work load. However, by reducing the screening appointment time, altering working patterns and purchasing a single extra workstation, the impact to the service has been minimised. Potential difficulties do arise in the event of sickness or leave.

